# Sulfated Hyaluronan Drives Cell Adaptation and Matrix Composition in Advanced 3D Breast Cancer Cell Models

**DOI:** 10.3390/cells15181687

**Published:** 2026-09-17

**Authors:** Christos Koutsakis, Katerina Mineschou, Konstantinos Spanopoulos, Sylvia Mangani, Marco Franchi, Evgenia Karousou, Zoi Piperigkou, Nikos K. Karamanos

**Affiliations:** 1 Biochemistry, Biochemical Analysis and Matrix Pathobiology Research Group, Laboratory of Biochemistry, Department of Chemistry, University of Patras, 26504 Patras, Greece; ckoutsakis@upatras.gr (C.K.); up1086993@upatras.gr (K.M.); up1087032@upatras.gr (K.S.); sylvia.mangani@upatras.gr (S.M.); 2 Department for Life Quality Studies, University of Bologna, 47921 Rimini, Italy; marco.franchi3@unibo.it; 3 Dipartimento di Medicina e Chirurgia, Universitá Degli Studi Dell’Insubria, 21110 Varese, Italy; jenny.karousou@uninsubria.it

**Keywords:** hyaluronan, sulfated hyaluronan, extracellular matrix, breast cancer, 3D cell platforms, spheroids

## Abstract

**Highlights:**

**What are the main findings?**

- Sulfated hyaluronan (sHA) suppressed long-term spheroid growth and reduced cell spreading in highly invasive MDA-MB-231 breast cancer (BC) cells.
- sHA modulated key regulators of extracellular matrix (ECM) remodeling and hyaluronan (HA) metabolism, including MMP-14, CEMIP, HAS2 and HYAL2.

**What are the implication of the main finding?**

- Chemical sulfation markedly alters the biological activity of HA with more profound effects in the aggressive triple-negative BC (TNBC) model.
- These findings support sHA as a promising ECM-targeting strategy and highlight the value of three-dimensional (3D) models for evaluating its antitumoral potential.

**Abstract:**

Hyaluronan (HA), a major extracellular matrix (ECM) glycosaminoglycan, plays a key role in breast cancer progression. Although native HA lacks sulfate groups, chemically modified sulfated hyaluronan (sHA) has demonstrated promising antitumor activity. Previous work from our group showed that sHA alters cellular functions and modulates ECM-related gene expression in triple-negative breast cancer (TNBC) cells. The aim of this study was to investigate the effects of low-molecular-weight HA (50 kDa) and its sulfated derivative of the same molecular weight, sHA, in MDA-MB-231 and MCF-7 breast cancer cells using advanced 3D cell culture models. Gene expression analyses focused on ECM components, including HA receptors and matrix metalloproteinases (MMPs) that were evaluated. The 3D cell morphology was examined using scanning electron microscopy (SEM). Spheroid growth and expression profiles linked to ECM remodeling and invasiveness were also assessed. Notably, sHA significantly inhibited 3D spheroid growth, reduced cell spreading in a cell line-dependent manner and influenced the expression of genes correlated with ECM remodeling and HA synthesis. These findings emphasize the importance of 3D models for studying ECM-driven breast cancer progression, further supporting sHA as a potential therapeutic modulator.

## 1. Introduction

Breast cancer (BC) encompasses a phenotypically diverse group of diseases and is the leading cause of death in women worldwide [1]. BC progression is closely correlated with cell–cell interactions within the tumor microenvironment (TME) that are driven by alterations in the composition of the extracellular matrix (ECM). The ECM is a dynamic and complex network composed of several macromolecules, such as proteoglycans/glycosaminoglycans (PGs/GAGs), collagens, laminins, elastin, hyaluronan (HA) and several matrix effectors [2]. The plethora of these macromolecular components is responsible for several cellular functions, including cell proliferation, migration, invasion and survival, that are linked to BC progression [3]. During the last few decades, several lines of evidence have identified the ECM as a highly dynamic structural and functional biomolecular network, involved in several stages of cancer dissemination and progression. Among them, epithelial-to-mesenchymal transition (EMT) is a key process in cancer progression characterized by the loss of epithelial features, including E-cadherin, and the acquisition of mesenchymal traits, such as Slug, which favor enhanced cell motility and migration [4].

Among the various GAGs, HA is ubiquitously distributed in ECMs, and upon binding with cell surface receptors and/or interactions with various hyaladherins, it exerts key biological functions in health and disease. Native HA is a non-sulfated, linear biopolymer with a variable number of the repeating disaccharide unit composed of D-glucuronic acid (GlcA) and N-acetyl-D-glucosamine (GlcNAc). Physiologically, HA contributes to the regulation of several biological processes, including embryonic development, tissue homeostasis and wound healing. Dysregulation in its production constitutes a characteristic of cancer cells, leading to the progression of the disease [5,6]. HA can be synthesized by three transmembrane enzymes (called HA synthases, HAS), HAS1, HAS2 and HAS3, that differ in their enzymatic activity, as HAS1 and HAS2 generally synthesize high-molecular-weight (HMW) HA while HAS3 synthesizes shorter fragments of HA [7]. The fragmentation of HA into low-molecular-weight (LMW) HA is mediated by the action of hyaluronidases (HYALs) [6]. HA exerts its biological effects primarily through interactions with cell surface receptors, particularly CD44 and the receptor for HA-mediated motility (RHAMM), thereby modulating signaling pathways involved in cancer cell migration, proliferation and invasion [8]. Taking into consideration that HA is the only GAG that is not sulfated, many research groups have tried to introduce sulfate groups into HA structures [5]. Previous results of our group have clearly demonstrated that sulfated HA (sHA) has antitumor effects, as it can decrease cancer cell proliferation, invasion and migration, influence the gene expression of several ECM molecules and inhibit tumor growth in several BC cell lines with distinct gene expression profiles [9]. Despite these promising findings, the effects of sHA on BC cells within more representative three-dimensional (3D) models remain insufficiently explored.

To bridge the gap between two-dimensional (2D) and 3D cultures, interest in using more representative models has emerged. Conventional 2D models fail to accurately reproduce the complexity of the TME, as they provide limited cell–cell and cell–ECM interactions and do not recapitulate the spatial gradients of oxygen and nutrients found in vivo. In contrast, 3D cell models better mimic the native tumor architecture by preserving cell–cell and cell–ECM interactions, establishing physiologically relevant oxygen and nutrient gradients and reproducing the cellular heterogeneity observed in tumors [10].

Therefore, the present study aims to investigate the biological effects of LMW HA (50 kDa) and chemically modified sHA on BC progression using 3D advanced cell culture models. MDA-MB-231 cells were used as a representative triple-negative BC (TNBC) model, and MCF-7 cells as a Luminal A model, respectively. Comparing these two models allows the assessment of whether HA-mediated responses are influenced by tumor aggressiveness and molecular subtype. Particular emphasis was placed on ECM remodeling, HA receptor expression, EMT-associated markers, matrix metalloproteinases (MMPs), spheroid growth and cellular morphology.

## 2. Materials and Methods

### 2.1. Cell Lines and Culture Conditions

MDA-MB-231 (HTB-26) and MCF-7 (HTB-22) cells were obtained from the American Type Culture Collection (ATCC, Manassas, VA, USA) and were maintained in Dulbecco’s modified Eagle’s medium (DMEM; LM-D1110/500, Biosera, Cholet, France). The growth medium contained 10% FBS (FBS; 10-FBS-16F, Capricorn Scientific, Düsseldorf, Germany), 1.0 mM sodium pyruvate, 2 mM L-glutamine, and antimicrobial agents (100 IU/mL penicillin, 100 µg/mL streptomycin, 10 µg/mL gentamycin sulfate, 2.5 µg/mL amphotericin B). Cell cultures were incubated at 37 °C in a humidified environment with 5% CO_2_.

### 2.2. HA and sHA Fragments

Low-molecular-weight HAan (50 kDa) and sHA (50 kDa, degree of sulfation 2.6, corresponding to 2–3 sulfate groups per disaccharide unit) were provided by Fidia Farmaceutici S.p.A. (Abano Terme, Italy). Stock solutions (2 mg/mL) were prepared in phosphate-buffered saline (PBS; LM-S2041, Biosera, France). For treatments, cells were starved for 16–20 h in serum-free DMEM, and then incubated with 200 μg/mL HA or sHA for 24 h.

### 2.3. 3D Spheroid Formation and Treatment

Spheroids were generated by seeding 10,000 cells/well in 96-well ultra-low-attachment plates (Sphera Low-Attachment Surface, Thermo Fisher Scientific, Waltham, MA, USA). Cells were incubated for 72 h without medium changes to allow spheroid formation. Spheroids were then starved in serum-free DMEM for 16–20 h, followed by 24 h treatment with 200 μg/mL 50 kDa HA or sHA. Spheroids treated with sHA or HA were monitored for 10 days with half-medium change every 2 days.

### 2.4. Spheroid Imaging and Morphometric Analysis

Spheroids were imaged using an Olympus BX51 phase-contrast microscope (Olympus, Tokyo, Japan) equipped with a digital camera. The spheroid area was quantified using ImageJ software (NIH, Bethesda, MD, USA). For growth size analysis, spheroids were harvested and dissociated, and viable cells were counted using a LUNA automated cell counter (Logos Biosystems, Villeneuve d’Ascq, France). Spheroid circularity was calculated in ImageJ software (version 1.52p, Launcher Symmetry Software, LOCI, University of Wisconsin, WI, USA) as 4π × Area/Perimeter^2^.

### 2.5. Scanning Electron Microscopy (SEM) of Spheroids

Spheroids were processed for scanning electron microscopy (SEM) according to a previously established protocol [11], with minor modifications. Briefly, samples were fixed using Karnovsky’s fixative, followed by osmium tetroxide post-fixation, graded ethanol dehydration and critical-point drying. The dried specimens were subsequently sputter-coated with gold–palladium and examined using a Philips 515 scanning electron microscope (Philips, Amsterdam, The Netherlands) at the CRIETT Center, University of Insubria (Varese, Italy).

### 2.6. Analysis of Spheroid Dissemination

Spheroid dissemination was assessed based on a previously described protocol [9], adapted here for 3D spheroid cultures. Following treatment with 50 kDa HA or sHAs (200 μg/mLs, 24 h), individual spheroids were placed onto type I collagen gel (1 mg/mLs). Images were acquired at 0 and 24 h, and cell spreading from the spheroid was quantified using Fiji (ImageJ, version 1.52p, Launcher Symmetry Software, LOCI, University of Wisconsin, WI, USA).

### 2.7. Immunofluorescence

Immunofluorescence staining of breast cancer (BC) spheroids was performed in 96-well ultra-low-attachment plates, based on a previously described immunofluorescence protocol with appropriate modifications [9]. Following a 24 h exposure to 50 kDa HA or sHAs (200 μg/mL), spheroids were processed for immunofluorescence and incubated overnight at 4 °C with mouse anti-CD44 (Hermes-3, 1 μg/mL, ab254530, Abcam, Waltham, MA, USA). Fluorescent secondary antibody labeling was subsequently performed, followed by DAPI nuclear counterstaining and fluorescence imaging. The samples were rinsed three times with PBS before incubation with Alexa Fluor 488-conjugated goat anti-mouse (diluted 1:500, Biotium, Fremont, CA, USA) for 3 h at room temperature. Following another series of three PBS washes, ProLong™ Gold Antifade Mountant with DAPI (Thermo Fisher Scientific, USA) was used for nuclear visualization. Confocal fluorescence images were subsequently acquired with a Leica TCS SP8 confocal laser-scanning microscope (Leica Microsystems, Wetzlar, Germany).

### 2.8. RNA Isolation, cDNA Synthesis and Real-Time qPCR

For total RNA isolation from 3D cell cultures, cell-derived spheroids were cultured for 72 h, starved for 16–20 h and then treated with HA or sHA for 24 h at a final concentration of 200 μg/mL. A total of 48 spheroids per condition (control, 50 kDa, and sHA), each seeded at a density of 15,000 cells, were collected for subsequent RNA extraction. Afterwards, spheroids were collected using a 1000 μL pipette tip and total RNA was isolated using the NucleoSpin^®^RNA Plus Kit (740984, Macherey-Nagel, Dueren, Germany), following the manufacturer’s instructions. The RNA concentration was determined spectrophotometrically at 260 nm, while the sample quality was evaluated based on the 260/280 and 260/230 absorbance ratios. RNA obtained from both 2D and 3D cultures was converted into cDNAs with the PrimeScript RT Reagent Kit (Perfect Real Time, RR037A, Takara Bio Inc., Goteborg, Sweden). Quantitative real-time qPCR was subsequently carried out following the supplier’s recommendations using Rotor Gene Q (Qiagen, Germantown, MD, USA). Non-template controls (NTCs) were incorporated into each qPCR run to monitor potential contamination, whereas melt curve analyses were used to verify amplification specificity. Additionally, a melting curve analysis was performed to detect the Maxima SYBR Green/ROX qPCR Master Mix (2X) enzyme (Thermo Fisher Scientific, Waltham, MA, USA). Cycle (Ct) values were determined by applying a fluorescence threshold above the background signal within the exponential phase of amplification. Relative transcript levels were quantified by the 2^−∆∆Ct^ approach using *18s rRNA* and *ACTB* as reference genes for normalization. Expression changes are reported as arbitrary units relative to the corresponding control. The genes analyzed and the primer sequences employed for real-time qPCR are presented in Table 1.

### 2.9. Statistical Analysis

Statistical significance was determined using unpaired two-tailed Student’s t-tests and/or one-way ANOVA with Tukey’s post hoc test. Significance levels: * *p* ≤ 0.05, ** *p* ≤ 0.01, *** *p* ≤ 0.001. Statistical analysis in spheroid growth experiments was performed by two-way ANOVA to estimate statistical differences in the mean values ±SD between the different treated groups. Reported values are expressed as the mean ± standard deviation (SD) of experiments in triplicate. Graphical representations were performed using GraphPad Prism version 8.0.1 (GraphPad Software, San Diego, CA, USA).

## 3. Results

### 3.1. sHA Modulates Surface Structure and Cellular Organization of Breast Cancer Spheroids

**Figure 1:**
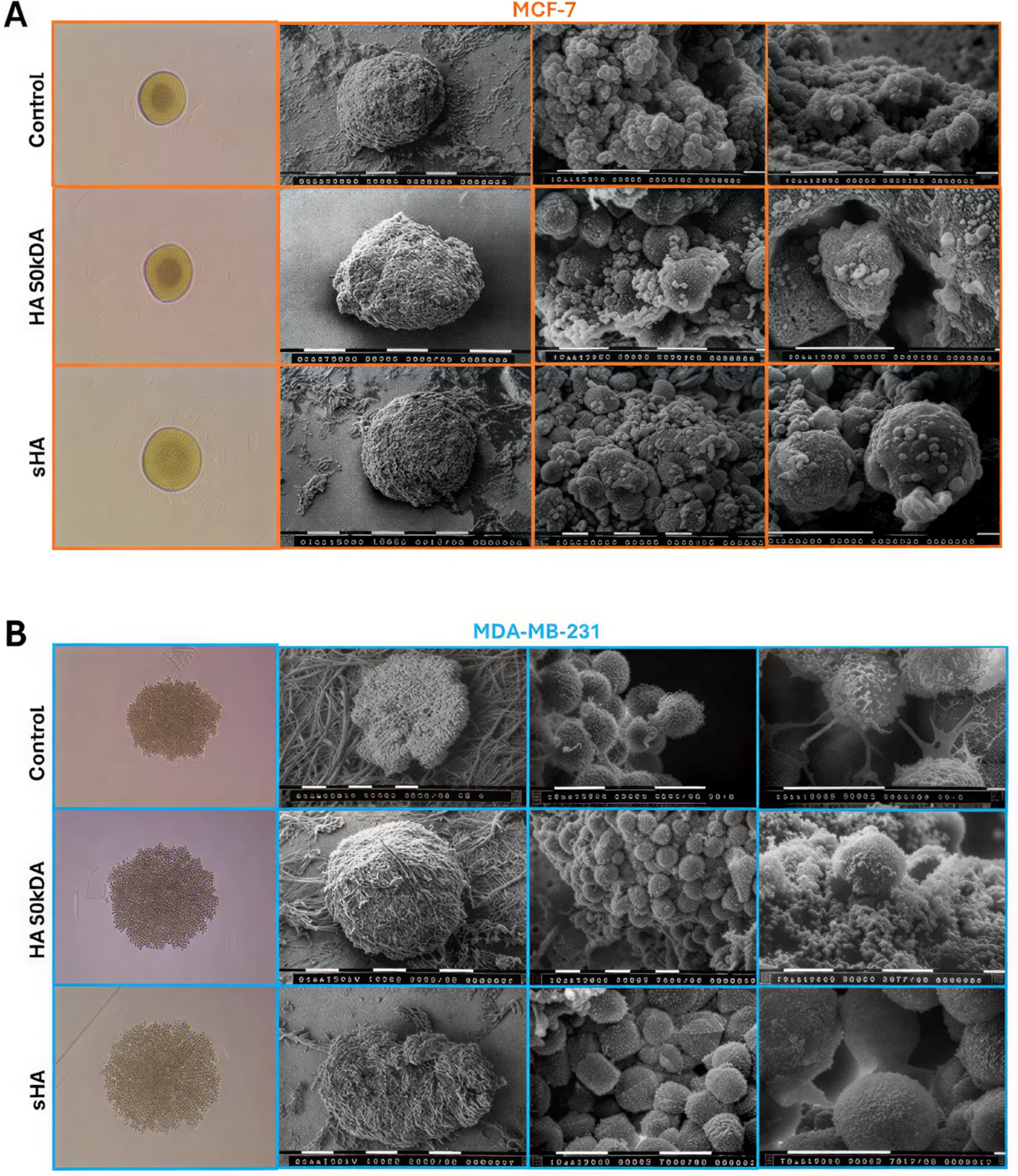
Evaluation of the effect of 50 kDa HA and sHA on the morphology of MCF-7 (**A**) and MDA-MB-231 (**B**) spheroids as assessed by scanning electron microscopy (SEM). Spheroids were treated with 50 kDa HA or sHA at a final concentration of 200 μg/mL for 24 h. Images in the left panels were acquired at 10× magnification. Representative SEM images are presented at increasing magnifications.

SEM was employed to examine the surface ultrastructure and overall morphology of BC spheroids following treatment with HA 50 kDa and sHA. This technique provides high-resolution visualization of spheroid architecture. MCF-7 spheroids formed compact, round aggregates with well-defined borders and a dense, dark core visible by day 3. This morphology is consistent with the epithelial, less invasive nature of Luminal A cells. Small intracellular gaps were observed throughout the spheroids, allowing nutrient and oxygen diffusion. However, treatment with either 50 kDa HA or sHA did not induce detectable changes in spheroid morphology or cellular organization compared to the control group (Figure 1A). In contrast, MDA-MB-231 spheroids exhibited loose, irregular architecture with less compact organization and less-defined borders, reflecting the mesenchymal, invasive phenotype of TNBC cells. MDA-MB-231-derived spheroids contained discrete intercellular spaces, again for the exchange of nutrients and oxygen. Direct cell–cell communication is conducted through fine membranous nanotubes (Figure 1B). In comparison with the control group, both the HA of 50 kDa and sHA-treated spheroids exhibited markedly fewer membrane protrusions. Moreover, cells exposed particularly to sHA appeared more flattened and closely apposed.

### 3.2. sHA Suppresses Long-Term Spheroid Expansion

**Figure 2:**
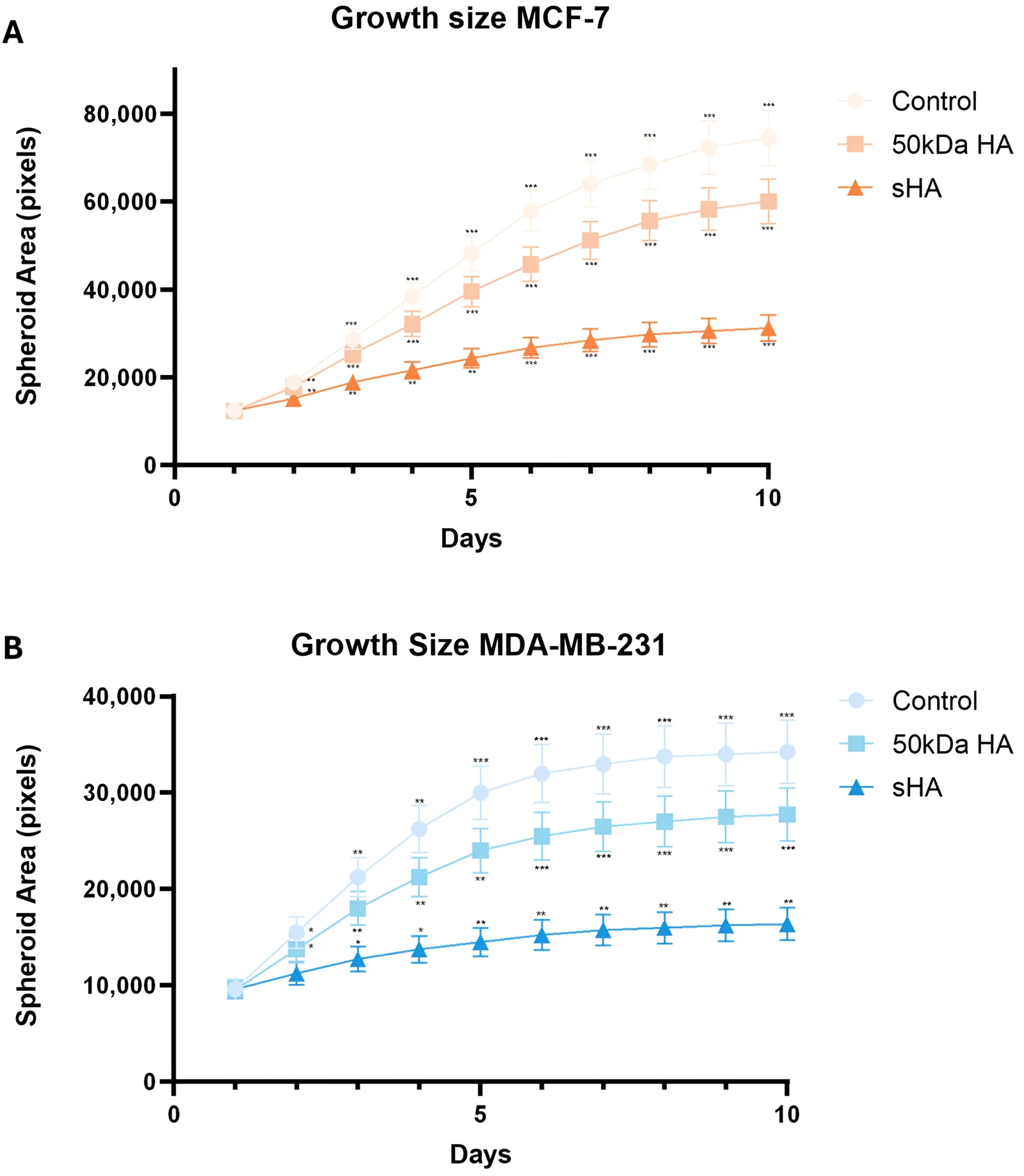
Ten-day growth kinetics of MCF-7 and MDA-MB-231 spheroids. Quantitative graph showing spheroid area for MCF-7 (**A**) and MDA-MB-231 (**B**) over 10 days. Data are mean ± SD versus day 1. One asterisk (*) indicates a statistically significant difference (*p* ≤ 0.05), two asterisks (**) indicate a statistically significant difference (*p* ≤ 0.01) and three asterisks (***) indicate statistically significant differences (*p* ≤ 0.001) compared to the untreated cells (control).

Ten-day growth curves revealed sustained sHA-mediated suppression of spheroid expansion. MCF-7 control spheroids progressively increased in area from day 1 to day 10 (approximately 3-fold expansion). sHA-treated MCF-7 spheroids showed reduced growth kinetics, with significant differences from day 3 onward (*p* < 0.01 at day 10) (Figure 2A). MDA-MB-231 control spheroids exhibited more rapid initial expansion but reached a plateau after day 5, likely due to nutrient limitations in the loose, irregular spheroids. Strikingly, sHA-treated MDA-MB-231 spheroids showed minimal expansion beyond day 3, maintaining a small, compact size throughout the 10-day culture period (*p* < 0.001 compared to control at day 10) (Figure 2B). These data indicate that sHA not only reduces initial spheroid growth but also limits long-term expansion capacity.

### 3.3. sHA Reduces Cell Spreading of Invasive Breast Cancer Cells on Extracellular Matrix Substrates

**Figure 3:**
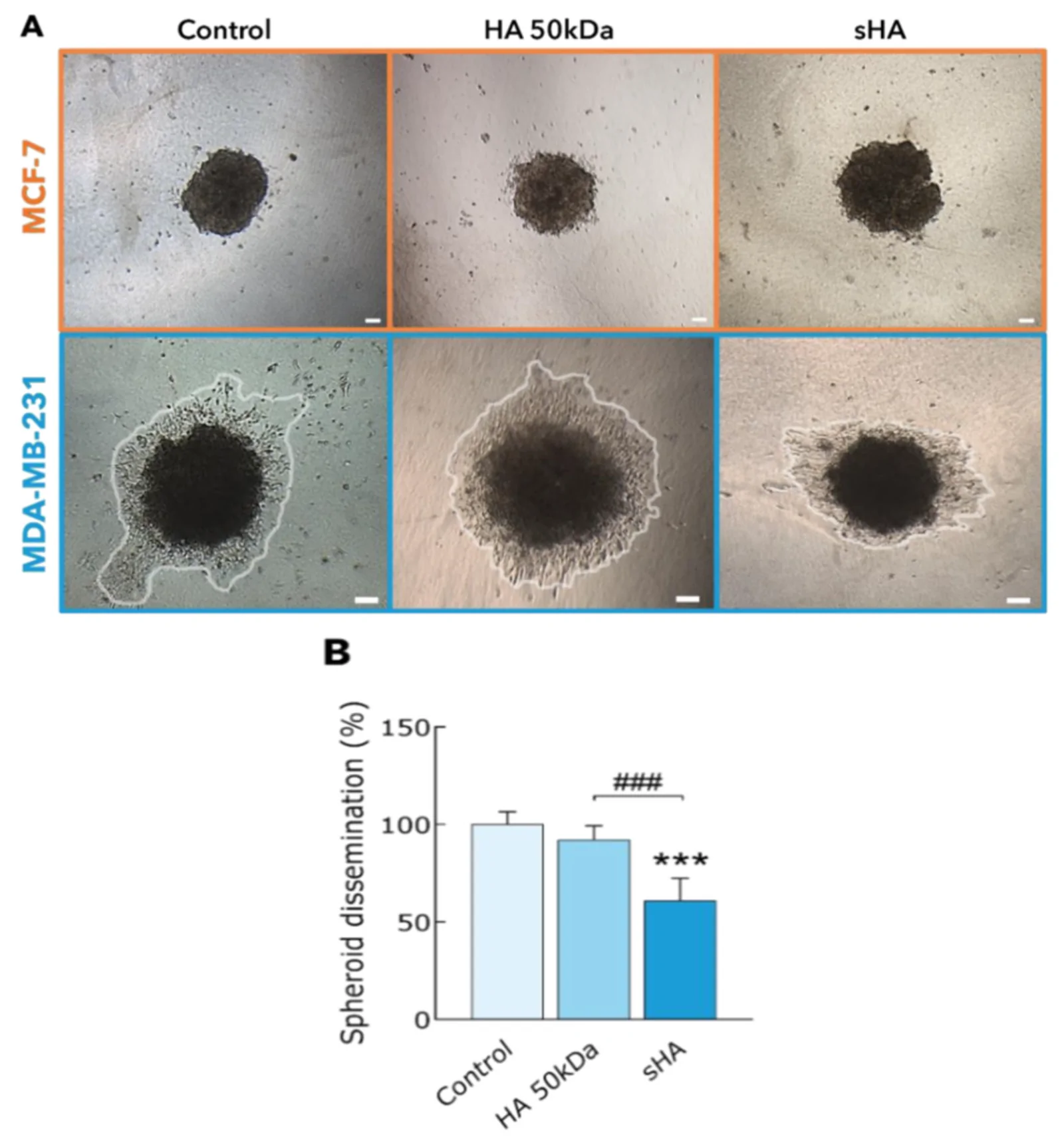
Evaluation of the effects of 50 kDa HA and sHA on the spreading of breast cancer spheroids in a three-dimensional type I collagen matrix. Spheroids were exposed to the indicated HA derivatives for 24 h at a final concentration of 200 μg/mL before being transferred onto collagen type I gels. (**A**) Representative phase-contrast micrographs illustrating the dissemination of MCF-7 and MDA-MB-231 spheroids after 24 h of culture on collagen. (**B**) Quantification of cell spreading in MDA-MB-231. Data are mean ± SD. Asterisks (***) indicate statistically significant differences compared with the untreated control group (*p* ≤ 0.01), whereas hash symbols (###) denote statistically significant differences between the treatment groups (*p* ≤ 0.01). Scale bar = 100 μm.

To gain further insights into the influence of sHA on the invasive behavior of BC spheroids, a spheroid spreading assay was carried out using a type I collagen matrix. Following treatment with either 50 kDa HA or sHA, spheroids were carefully transferred onto collagen type I gels, and the extent of cellular outgrowth from the spheroid core was assessed by measuring the dissemination area at baseline (0 h) and after 24 h (Figure 3A).

Analysis of the spreading assay revealed distinct responses among the two examined BC cell lines. Particularly, MCF-7 spheroids exhibited limited spreading under all experimental conditions. Although the representative images suggest some variability in cellular outgrowth following treatment, no quantitative conclusions regarding treatment-induced changes in MCF-7 spreading can be drawn from the present analysis. Conversely, the highly invasive MDA-MB-231 spheroids responded differently to sHA treatment. After 24 h, sHA significantly restricted spheroid spreading by approximately 40%, compared with untreated controls (Figure 3B). These findings suggest that sHA impairs the ability of aggressive BC cells to spread within a collagen-rich matrix, whereas the effect of sHA on MCF-7 cell spreading could not be quantitatively established in the present analysis.

### 3.4. sHA Modulates Gene Expression in 3D Spheroids

**Figure 4:**
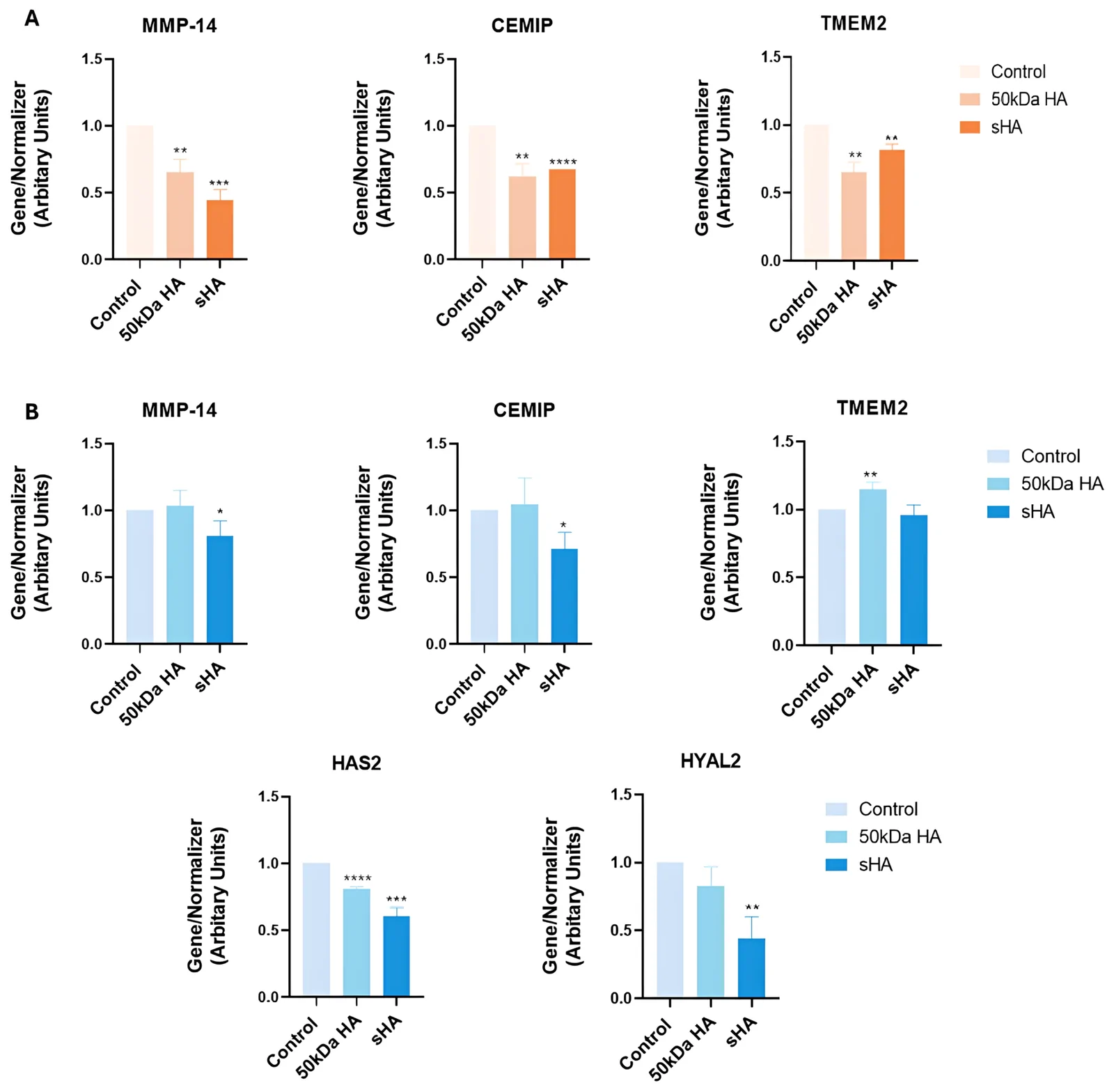
Effects of 50 kDa HA and sHA on the expression of hyaluronan-related genes. Relative mRNA expression of several genes was determined by qPCR following 24 h treatment with 50 kDa HA and sHA for (**A**) MCF-7 and (**B**) MDA-MB-231. Gene expression levels were normalized to the reference gene and are presented relative to the control group. Data are shown as mean ± SD. One asterisk (*) indicates a statistically significant difference (*p* < 0.05), two asterisks (**) indicate a statistically significant difference (*p* < 0.01), three asterisks (***) indicate statistically significant differences (*p* < 0.001) and four asterisks (****) indicate statistically significant differences (*p* < 0.0001)compared to the untreated cells (control).

To investigate whether HA derivatives affect the expression of genes involved in HA metabolism and ECM remodeling, qPCR analysis was performed following treatment with 50 kDa HA and sHA in MCF-7-derived spheroids (Figure 4A) and in MDA-MB-231-derived spheroids (Figure 4B). Specifically, the expression of *MMP-14*, a key mediator of ECM degradation and tumor invasion, was assessed together with *CEMIP*, an HA-depolymerizing protein implicated in BC progression. In addition, *TMEM2* and *HYAL2*, two major hyaluronidases responsible for HA degradation and turnover, were evaluated, while *HAS2*, the predominant HAS in BC, was analyzed to determine whether HA derivatives influence endogenous HA biosynthesis.

Both cell lines exhibited reduced *MMP-14* and *CEMIP* expressions following sHA treatment. However, the inhibitory effect was more pronounced in MCF-7-derived spheroids for *MMP-14*, whereas *CEMIP* expression was markedly reduced in MDA-MB-231-derived spheroids. In contrast, *TMEM2* expression displayed distinct responses between the two cell lines. While 50 kDa HA reduced *TMEM2* expression in MCF-7-derived spheroids, the same treatment increased *TMEM2* expression in MDA-MB-231-derived spheroids, which was abolished following sHA treatment, restoring expression to control levels.

Additional analysis in MDA-MB-231-derived spheroids demonstrated that sHA significantly downregulated both *HAS2* and *HYAL2* expression, whereas treatment with 50 kDa HA produced minor changes in *HYAL2*. *HAS2* was evaluated exclusively in MDA-MB-231 cells because publicly available transcriptomic data from The Human Protein Atlas indicate negligible basal expression in MCF-7 cells (0.1 transcripts per million, TPM) compared with MDA-MB-231 cells (8.4 TPM), suggesting a limited contribution of *HAS2* to HA synthesis in the less invasive phenotype. Although *HYAL2* is expressed in both cell lines (29.4 TPM in MCF-7 and 37.7 TPM in MDA-MB-231), its expression was examined only in MDA-MB-231 cells to provide a more comprehensive assessment of HA metabolism in the highly invasive model, where the most pronounced biological effects of sHA were observed.

### 3.5. sHA Does Not Affect the Localization of CD44 in Breast Cancer Spheroids

**Figure 5:**
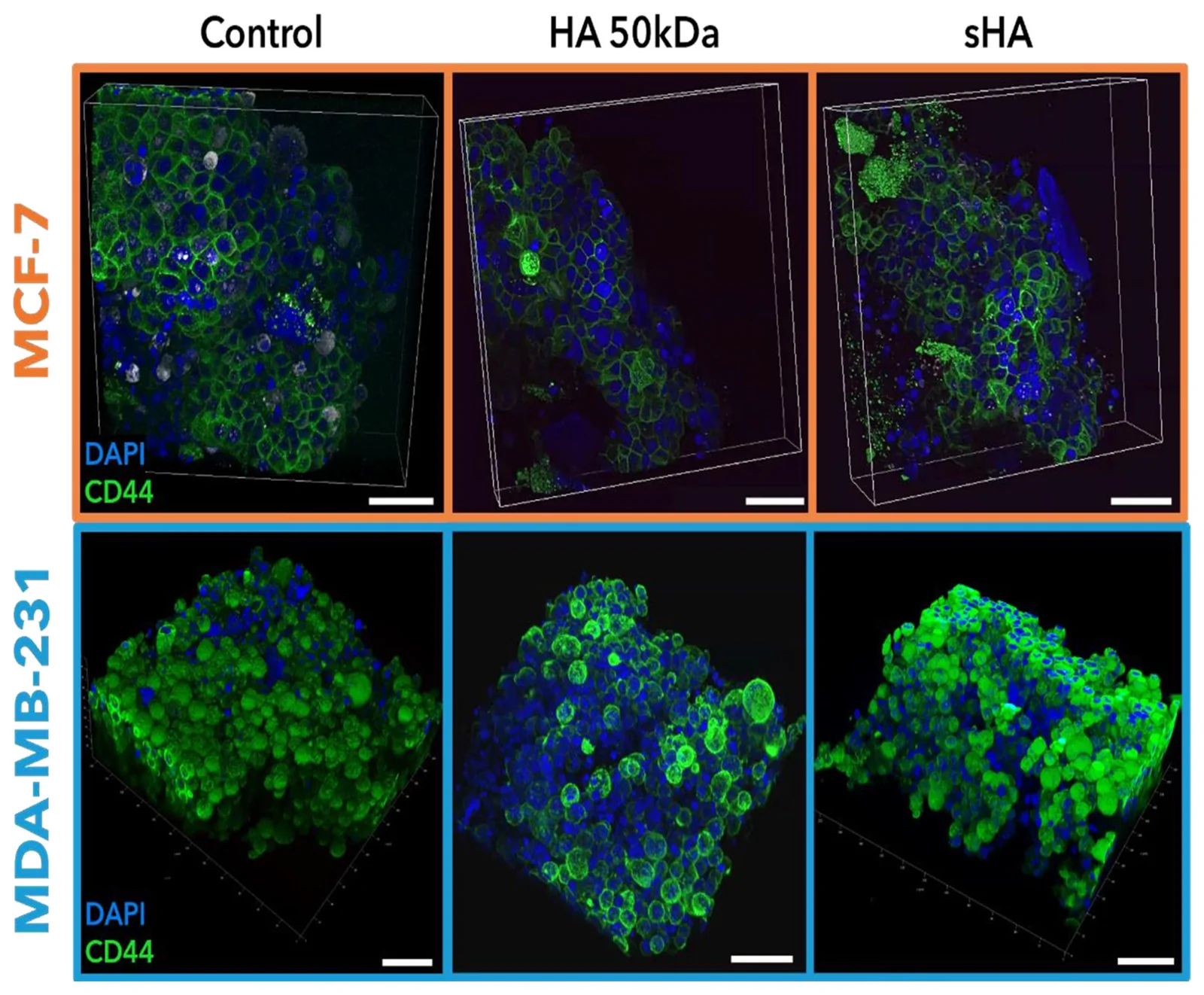
Three-dimensional reconstruction of representative regions of MCF-7 and MDA-MB-231 spheroids following immunofluorescence staining for the CD44 receptor (green). Spheroids were treated with 50 kDa HA or sHA at a final concentration of 200 μg/mL for 24 h. Cell nuclei are shown in blue. Scale bar: 50 μm.

As CD44 is the principal cell surface receptor for HA and mediates many of its effects on cell adhesion, migration and ECM remodeling, its localization was evaluated following treatment with 50 kDa HA and sHA. MCF-7- and MDA-MB-231-derived spheroids were immunostained using Hermes-3 anti-CD44 antibody. Qualitative immunofluorescence analysis revealed no apparent differences in the localization or distribution of CD44 among control, 50 kDa HA-treated and sHA-treated spheroids in either BC cell line (Figure 5), suggesting that neither treatment markedly altered CD44 localization under the experimental conditions.

## 4. Discussion

BC progression is strongly influenced by the dynamic interactions between tumor cells and the ECM, with HA representing one of the major ECM components regulating tumor cell behavior [12]. Since the biological activity of HA depends largely on its molecular size and chemical structure, understanding how modified HA derivatives affect BC cells is of particular interest for the development of novel therapeutic approaches. In the present study, we compared the effects of native LMW HA and chemically modified sHA using advanced 3D spheroid models of Luminal A (MCF-7) and TNBC (MDA-MB-231) BC cell lines. Overall, our findings demonstrated that sHA exerts a stronger biological effect than native HA, particularly in the highly invasive TNBC model.

3D spheroids better reproduce the structural organization, and cell–cell and cell–ECM communication observed in solid tumors than conventional 2D cultures. Consequently, they provide a more physiologically relevant platform for evaluating tumor progression and therapeutic responses [13,14]. The distinct morphology observed between MCF-7- and MDA-MB-231-derived spheroids is consistent with the intrinsic characteristics of these cell lines. MCF-7-derived spheroids remained compact and well organized, reflecting their epithelial phenotype, whereas MDA-MB-231-derived spheroids exhibited a loose architecture with irregular borders, consistent with their mesenchymal and highly invasive phenotype. These observations confirm that the selected 3D models successfully reproduce the biological heterogeneity of BC [15].

SEM analysis further demonstrated that sHA did not dramatically alter the overall spheroid architecture but induced subtle ultrastructure changes, particularly in MDA-MB-231-derived spheroids. The reduction in membrane protrusions and the appearance of flatter, more closely apposed cells suggest reduced cellular plasticity and weaker interactions with the surrounding matrix. Since membrane protrusions, such as filopodia, are essential for cell migration and invasion, their reduction may represent an early morphological indication of impaired metastatic potential [16]. These ultrastructural observations are consistent with the functional assays demonstrating reduced cell spreading following sHA treatment.

Long-term growth experiments further support the antitumor activity of sHA. Although both cell lines exhibited continuous spheroid expansion under control conditions, sHA markedly inhibited spheroid growth throughout a 10-day culture period. The inhibitory effect was particularly pronounced in MDA-MB-231-derived spheroids, suggesting that aggressive BC cells are more susceptible to the biological effects of sHA. The present study extended these observations by showing that this inhibitory activity is maintained in more physiologically relevant 3D models. It should be noted that changes in spheroid size do not necessarily reflect cytotoxicity and may also result from alterations in proliferation, cell organization or spheroid compaction. Therefore, further analyses of cell viability and cell death are required to establish the mechanisms underlying the observed changes in spheroid growth.

One of the most important findings of the present study is the significant reduction in spheroid spreading observed in MDA-MB-231-derived spheroids following sHA treatment. Cell spreading from tumor spheroids represents an early step of local invasion and metastatic spread. Therefore, the approximately 40% reduction in dissemination observed in collagen type I matrices suggests that sHA interferes with the ability of aggressive BC cells to invade collagen-rich ECM. In contrast, MCF-7-derived spheroids displayed minimal spreading regardless of treatment, consistent with their inherently low invasive potential. These observations indicate that the biological effects of sHA are more evident in highly invasive tumors, where HA-mediated signaling is more active [17].

qPCR analysis further supports this interpretation. The expression of genes involved in ECM remodeling and HA metabolism was differentially regulated by sHA. At the transcriptional level, sHA treatment was associated with reduced *MMP-14* in ECM degradation and tumor invasion [18] and *CEMIP* in HA depolymerization [19,20]; these transcriptional changes may be considered with reduced ECM remodeling activity. However, as protein abundance and functional activity were not assessed in the present study, the functional consequences of these changes cannot be directly inferred from the qPCR data and require further validation.

Interestingly, *TMEM2* exhibited cell line-dependent regulation. While native HA transiently increased *TMEM2* expression in MDA-MB-231-derived spheroids, sHA abolished this response, whereas in MCF-7-derived spheroids, the effects were modest. These findings suggest that sulfation modifies the cellular response to HA rather than simply enhancing or suppressing HA signaling. The precise mechanisms regulating *TMEM2* expression remain to be elucidated but may reflect differences in HA turnover between epithelial and mesenchymal BC cells.

Additional analysis of HA metabolic enzymes demonstrated that sHA significantly reduced *HAS2* and *HYAL2* expression in MDA-MB-231-derived spheroids. *HAS2* is considered the principal HAS in aggressive BC and contributes to excessive HA accumulation within the TME [21]. Therefore, the observed reduction in *HAS2* mRNA expression may indicate transcriptional regulation of endogenous HA synthesis by sHA. Similarly, the decrease in *HYAL2* mRNA expression suggests a potential effect on pathways involved in HA turnover, as *HYAL2* contributes to HA degradation and the generation of bioactive LMW HA fragments associated with BC progression [6,22]. However, these findings reflect changes at the transcriptional level and do not establish corresponding alterations in protein abundance, enzymatic activity or HA synthesis and degradation. Protein-based and functional analyses will therefore be required to determine the biological consequences of these transcriptional changes.

Despite these molecular alterations, immunofluorescence analysis demonstrated no detectable changes in CD44 localization following treatment. CD44 is the major receptor mediating HA signaling in BC and regulates cell adhesion, migration and survival [23]. The absence of qualitative changes in receptor localization suggests that the biological effects of sHA are unlikely to result from redistribution of CD44 at the cell surface. Instead, sHA may influence downstream CD44 signaling or alter ligand–receptor interactions through its modified chemical structure. Further studies investigating receptor activation, phosphorylation-dependent signaling pathways and CD44 binding affinity will be necessary to clarify these mechanisms.

The present study has several limitations. Gene expression was evaluated at the mRNA level, and additional protein-based analyses are required to confirm the observed transcriptional changes. Furthermore, only two BC cell lines were examined and further studies should include additional molecular subtypes as well as patient-derived organoids [24]. In addition, the absence of a non-malignant BC cell model limits conclusions regarding the cancer selectivity of sHA. Further studies should therefore evaluate the effects of sHA in non-malignant BC cells to determine whether its growth-inhibitory effects preferentially affect malignant cells. The investigation of downstream signaling pathways, including CD44-RHAMM and PI3K/AKT-dependent mechanisms, will further clarify the therapeutic potential of sHA.

Overall, our findings demonstrated that chemical sulfation profoundly modifies the biological activity of HA. Rather than acting as a passive structural ECM component, sHA actively modulates HA metabolism, ECM remodeling and the invasive behavior of aggressive BC cells. These observations further support sHA as a promising ECM-targeted therapeutic strategy for limiting BC progression.

## 5. Conclusions

In conclusion, the present study demonstrates that chemically modified sHA exerts stronger biological effects than native 50 kDa HA in advanced 3D BC models. Compared with native HA, sHA inhibited long-term spheroid growth, reduced the spreading of highly invasive MDA-MB-231-derived spheroids and modulated the expression of genes involved in ECM remodeling and HA metabolism, including *MMP-14*, *CEMIP*, *HAS2* and *HYAL2*. These findings suggest that sHA disrupts HA homeostasis and limits ECM remodeling associated with BC progression. Notably, the effects of sHA were more pronounced in the aggressive MDA-MB-231 model than in the Luminal A MCF-7 model, highlighting the importance of tumor subtype in determining the cellular response to HA derivatives. Although CD44 localization remained unchanged, the observed molecular and functional alterations indicate that sHA may regulate HA-mediated signaling through mechanisms other than receptor redistribution. Overall, this study highlights the value of 3D spheroid models for investigating ECM-targeted therapies and further supports sHA as a promising therapeutic candidate for limiting BC progression. Further studies should investigate the downstream signaling pathways involved in sHA-mediated effects and validate these findings in more complex preclinical models.

## Figures and Tables

**Table 1:** Primer sequences used for real-time polymerase chain reaction analysis.

Gene	Primer Sequence (5′-3′)	Annealing T (°C)
*CEMIP*	F: CACTGTCTCGGCTACAGACC	60
	R: TCAGAGACCGAGTGATGGGT	
*TMEM2*	F: AGGCACCTGAGTTAACGGAC	60
	R: GAGGAAAGCAGGGGAGTGTC	
*HAS2*	F: TCGCAACACGTAACGCAAT	60
	R: ACTTCTCTTTTTCCACCCCATTT	
*MMP14*	F: CATGGGCAGCGATGAAGTCT	60
	R: CCAGTATTTGTTCCCCTTGTAGAAGTA	
*HYAL2*	F: CTAATGAGGGTTTTGTGAACCAGAATAT	60
	R: GCAGAATCGAAGCGTGGATAC	
*ACTB*	F: TCAAGATCATTGCTCCTCCTGAG	60
	R: ACATCTGCTGGAAGGTGGACA	
*18S rRNA*	F: CAGGTCTGTGATGCCCTTAGA	60
	R: GCTTATGACCCGCACTTACTG	

## Data Availability

The original contributions presented in this study are included in the article. Further inquiries can be directed to the corresponding authors.

## Abbreviations

The following abbreviations are used in this manuscript:

2D	Two-dimensional
3D	Three-dimensional
BC	Breast cancer
BSA	Bovine serum albumin
CD44t	Total CD44 isoform
CEMIP	Cell migration-inducing hyaluronidase
DAPI	4′,6-diamidino-2-phenylindole
DMEM	Dulbecco’s Modified Eagle Medium
ECM	Extracellular matrix
EMT	Epithelial-to-mesenchymal transition
GAG	Glycosaminoglycan
GlcA	D-glucuronic acid
GlcNAc	N-acetyl-D-glucosamine
HA	Hyaluronan/hyaluronic acid
HAS1/2/3	Hyaluronan synthase 1, 2, 3
HAS2-AS1	HAS2 antisense RNA 1
HMW	High-molecular-weight
LMW	Low-molecular-weight
MMP	Matrix metalloproteinase
MMP-14	Matrix metalloproteinase 14
PBS	Phosphate-buffered saline
PFA	Paraformaldehyde
PI3K	Phosphoinositide 3-kinase
PG	Proteoglycan
RHAMM	Receptor for hyaluronan-mediated motility
SEM	Scanning electron microscopy
sHA	Sulfated hyaluronan
SNAI2	Snail family transcriptional repressor 2 (Slug)
TMEM2	Transmembrane protein 2 (hyaluronidase)
TME	Tumor microenvironment
TNBC	Triple-negative breast cancer
TPM	Transcripts per million
